# Rehabilitation of the burn patient

**DOI:** 10.4103/0970-0358.70730

**Published:** 2010-09

**Authors:** Fiona Procter

**Affiliations:** Lead Occupational Therapist for Burns, Alder Hey Children’s Hospital, Liverpool, UK

**Keywords:** Burn rehabilitation, splintage, range of movements, post burn physiotherapy

## Abstract

Rehabilitation is an essential and integral part of burn treatment. It is not something which takes place following healing of skin grafts or discharge from hospital; instead it is a process that starts from day one of admission and continues for months and sometimes years after the initial event. Burns rehabilitation is not something which is completed by one or two individuals but should be a team approach, incorporating the patient and when appropriate, their family. The term ‘Burns Rehabilitation’ incorporates the physical, psychological and social aspects of care and it is common for burn patients to experience difficulties in one or all of these areas following a burn injury. Burns can leave a patient with severely debilitating and deforming contractures, which can lead to significant disability when left untreated. The aims of burn rehabilitation are to minimise the adverse effects caused by the injury in terms of maintaining range of movement, minimising contracture development and impact of scarring, maximising functional ability, maximising psychological wellbeing, maximising social integration

## INTRODUCTION

The rehabilitation for patients with burn injuries starts from the day of injury, lasting for several years and requires multidisciplinary efforts. A comprehensive rehabilitation programme is essential to decrease patient’s post-traumatic effects and improve functional independence.[1] However, while optimal treatment provision involves a multidisciplinary team approach, when this is not possible or when availability of therapists and support services are limited, all members of the burns team can take responsibility for their part in rehabilitation to maximise the benefit to the patient. While different professionals possess expertise in their own specialities, there are some simple and effective methods that can be utilised to help the patient reach their maximum functional outcome. It is the dedication of the individuals within the burn team and the commitment to caring for the patient and encouraging them to participate and engage fully in their rehabilitation, which can make such a difference to their long-term quality of life.

In this article, an effort is being made to share the basic aspects of burn rehabilitation and provide practical information, which can be followed by different professionals working within the speciality of burns (and can be taught to family members) to best help their patients.

## STAGES OF REHABILITATION

Rehabilitation of burns patients is a continuum of active therapy starting from admission. There should be no delineation between an ‘acute phase’ and a ‘rehabilitation phase’[2] as this idea can promote the inequality of a secondary disjointed scar management and/or functional rehabilitation team.[3] However, for the ease of following a pathway of patient care, the stages of rehabilitation have been divided into early stages and later stages of rehabilitation; although, it must be understood that there may be significant crossover between these two stages depending on the individual patient.

## EARLY STAGES OF REHABILITATION

Depending on the size and severity of the injury, the patient’s age and other pre-morbid factors, this stage can last from a few days to several months. The patient may be an inpatient or may be treated as an outpatient and is likely to be undergoing regular dressing changes, which are often painful and may also be a very frightening experience for the patient.

Regular pain relief is essential, in particular prior to all interventions such as change of dressing and exercise; this needs to be given in adequate time to take effect before commencing the procedure. The aim of analgesic drugs should be to develop a good baseline pain control to allow functional movement and activities of daily living to occur at any time during the day.[2] Inadequate pain relief in the early stages can result in a complete reluctance of the patient to participate in their rehabilitation in both the short and long term.

Early commencement of rehabilitation is the key to compliance with treatment and maximising long-term outcome. When the various aspects of rehabilitation are introduced as an integral part of care from day one, whether the patient is an in-patient or out-patient, they are easier for the patient to accept and follow rather than as an additional element to their care at a later date when contractures are already developing.

## REMEMBER TOMORROW MIGHT BE TOO LATE!

Patients may want to delay their rehabilitation until they feel better; however, every day without burn therapy intervention will make the eventual rehabilitation process more difficult and painful and may result in a poorer outcome. If windows are missed, they cannot be regained easily, since the inevitable sequelae of ever-increasing joint stiffness and tethered soft-tissue glide become more and more devastating with the passage of time.[4] Patients may try to refuse treatment as they are in pain and may not understand fully the impact of not participating in their rehabilitation; they therefore need the support and encouragement of the burn care professionals to help them through this difficult experience with the knowledge of how different their quality of life can be.

## CRITICAL CARE

It is essential that physical rehabilitation is commenced at day 1 of admission whether the patient is ambulant and well or on bed rest and immobile.

When a patient is admitted with severe burns, it is essential to reduce the risks, as far as possible, of further complications arising. Postural management of the patient by elevating the head and chest helps with chest clearance and reduces swelling of the head, neck and upper airway. In the early stages, significant oedema may be present particularly in the peripheries; poor positioning can lead to unnecessary additional morbidity which can be avoided. Elevation of all limbs affected is necessary in order to quickly reduce oedema; hands should be splinted or positioned and feet kept at 90 degrees, care and attention must also be given to the heel area which can quickly develop pressure. Legs should be positioned in a neutral position ensuring that patient is not externally rotating at the hips [Graph 1].

Patients who are unable to move should have passive movements completed to maintain range of movement (ROM) and prevent stiffness developing. If due to surgical intervention and skin grafting this is not possible on a daily basis, it may be achieved during change of dressings.

**Figure:**
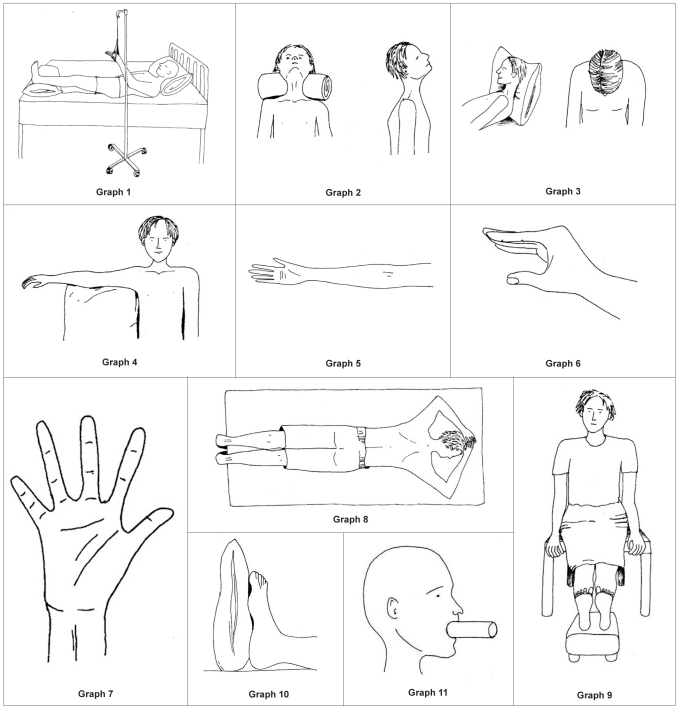


## PSYCHOLOGICAL IMPACT

It is important to remember that burn patients have often experienced a very frightening event leading to their burn injury and that the hospital experience itself can be frightening. Patients and family members may be experiencing significant feelings of guilt, anger and despair; they may also be having nightmares and flashbacks of the event. While professionals may treat many people in one day, the experience for each individual patient is personal and their experience can impact on their mental wellbeing and readiness to participate in their treatment. It is important that the patient is given comfort and reassurance that they are safe. Taking the time to listen to the patient’s concerns, demonstrating genuine empathy and compassion, providing adequate information and answering their questions can often go a long way to alleviating fears, which in turn can ease the treatment process for both patient and professional.

## ANTI-CONTRACTURE POSITIONING

**Figure 1:**
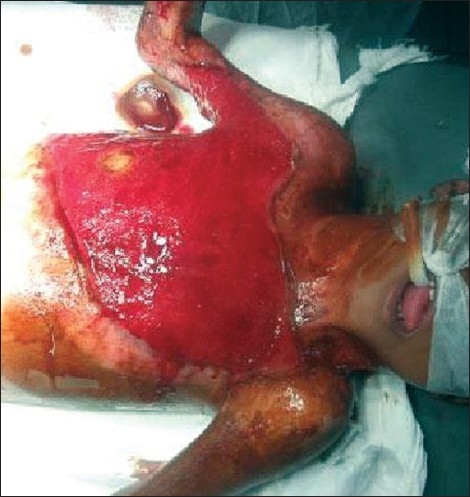
Burn wound not being nursed in anti contracture position with impending neck and axillary contracture

Anti-contracture positioning and splinting must start from day one and may continue for many months post-injury. It applies to all patients whether they have been skin grafted or not. Positioning is important to influence tissue length by limiting or inhibiting loss of ROM secondary to the development of scar tissue.[5] Patients rest in a position of comfort; this is generally a position of flexion and also the position of contracture. Wounds start the healing process almost as soon as they occur and a major part of this process is wound contracture as demonstrated in the photograph below [Figure 1].

Without ongoing advice and help with positioning, the patient will continue to take the position of contracture and can quickly lose ROM in multiple joints. Once contracture starts to develop it can be a constant battle to achieve full movement, so preventative measures to minimise contracture development are necessary. Early compliance is essential to ensure the best possible long-term outcome and also to ease pain and assist with exercise regimes.

Patients need to adhere to a positioning regime in the early stages of healing and this takes teamwork and dedication. The patient requires encouragement to maintain anti-contracture positioning all of the time (except for when carrying out exercise programmes and functional activities), not just during the time when the therapist is available to complete their ward round; support of the patient’s family can be crucial at this stage to assist the patient in maintaining the correct position when the hospital staff are not available. Educating the patient and family so they have a good understanding as to the benefits of participating in therapy is essential and getting the family on board at this early stage also means that they are more prepared to assist the patient on their return home.

It is crucial not to overlook patients who have relatively minor burns as they may also develop serious and debilitating contractures, which could be easily avoided by positioning, splinting and exercises.

When burns occur to the flexor aspect of a joint or limb the risk of contracture is greater. This is due to the position of comfort being a flexed position; also the flexor muscles are generally stronger than the extensors so should a burn occur to the extensor aspect, patients can use the strength of the flexors to stretch the particular area. The flexed position is the position of function for example clasping the hand, forward flexion of the shoulder and flexing the neck. The aims of anti-contracture positioning are to counteract this natural tendency towards flexion as demonstrated in the table below.

**Table 1:** Common post burn contractures and the respective anti contracture position of nursing

*Area burnt*	*Contracture/ difficulty experienced*	*Anti-contracture position*
Front of neck	Neck flexion. The chin is pulled towards the chest reducing neck movement. Contours of the neck are lost [Figure 2]	Neck in extension. No pillow behind head, roll behind neck. Head tilted back in sitting [Graph 2]
Posterior neck	Neck extension and other neck movements [Figure 3]	Sitting with head in flexion. Lying with pillows behind the head [Graph 3]
Axillas or anterior and posterior axilliary fold	Limited abduction, protraction when burns also to chest [Figure 4]	Lying and sitting - arms abducted to 90 degrees supported by pillows or foam blocks between chest and arms. Figure of eight bandaging or strapping to provide stretch across chest [Graph 4].
Front of elbows	Elbow flexion [Figure 5]	Elbow extension [Graph 5]
Back of hands	Metacarpalphalangeal (MCP)	Wrist - 30–40 degrees extended, MCPs 60-70 degrees flexion, IP joints in extension, thumb mid-palmar radial abduction [Graph 6]
	Hyperextension, interphalangeal (IP)	
	Flexion	
	Adduction of thumb	
	Wrist flexed [Figure 6]	
Palm of hand	Fingers adducted and flexed, palm pulled inwards [Figure 7]	Wrist extended, minimal MCP flexion, fingers extended and abducted. [Graph 7]
Groin (hip)	Hip flexion	Lie in prone with legs extended. Limit sitting and side lying. Supine lying with legs extended, no pillow under knees [Graph 8]
	Hip adduction [Figure 8]	
Back of knee	Knee flexion [Figure 9]	Legs extended in lying and sitting [Graph 9]
Feet	Feet are complex structures and can be pulled in different directions by healing tissues preventing normal mobility [Figure 10] [Figure 11]	Ankles at 90 degrees – use pillows to maintain position. Encourage sitting with feet flat on floor as long as no oedema present [Graph 10].
Face	The face can be effected in various different ways including inability to open or closer mouth fully and inability to close eyes fully	Regular change of facial expression and stretching regime required. A well-padded tube can be inserted into the mouth to combat mouth contracture [Graph 11]

**Figure 2:**
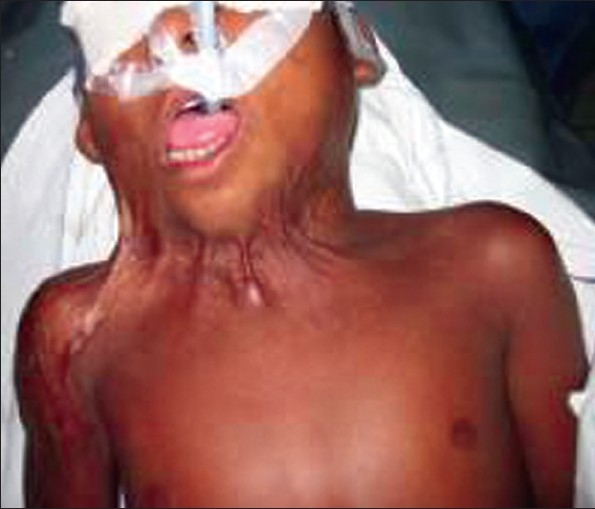
The flexion contracture of the neck can be avoided by having a pillow under the shoulder and nursing with neck in extension. There should be no pillow under the head

**Figure 3:**
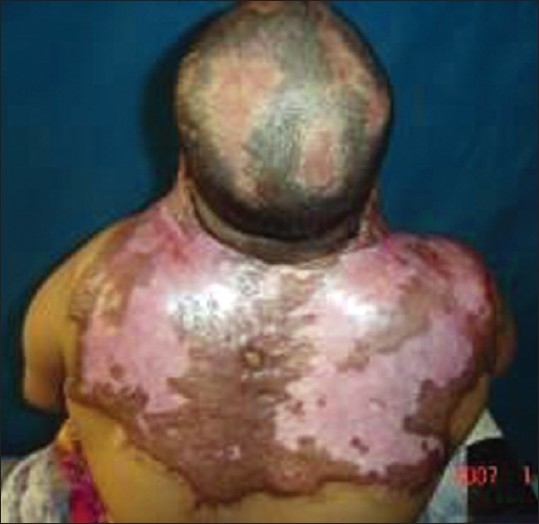
This extension contracture of the neck can be avoided by sitting with head in flexion ane lying with pillows behind the head

**Figure 4:**
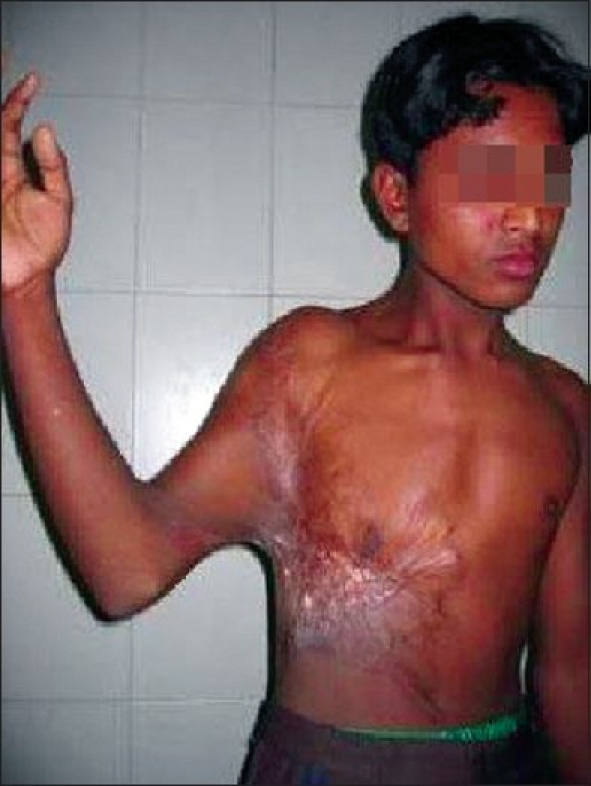
This axillary contracture can be prevented by lying and sitting with arms abducted at 90 degrees supported by pillows or foam blocks between chest and arms and figure of eight bandaging or strapping to provide stretch across chest

**Figure 5:**
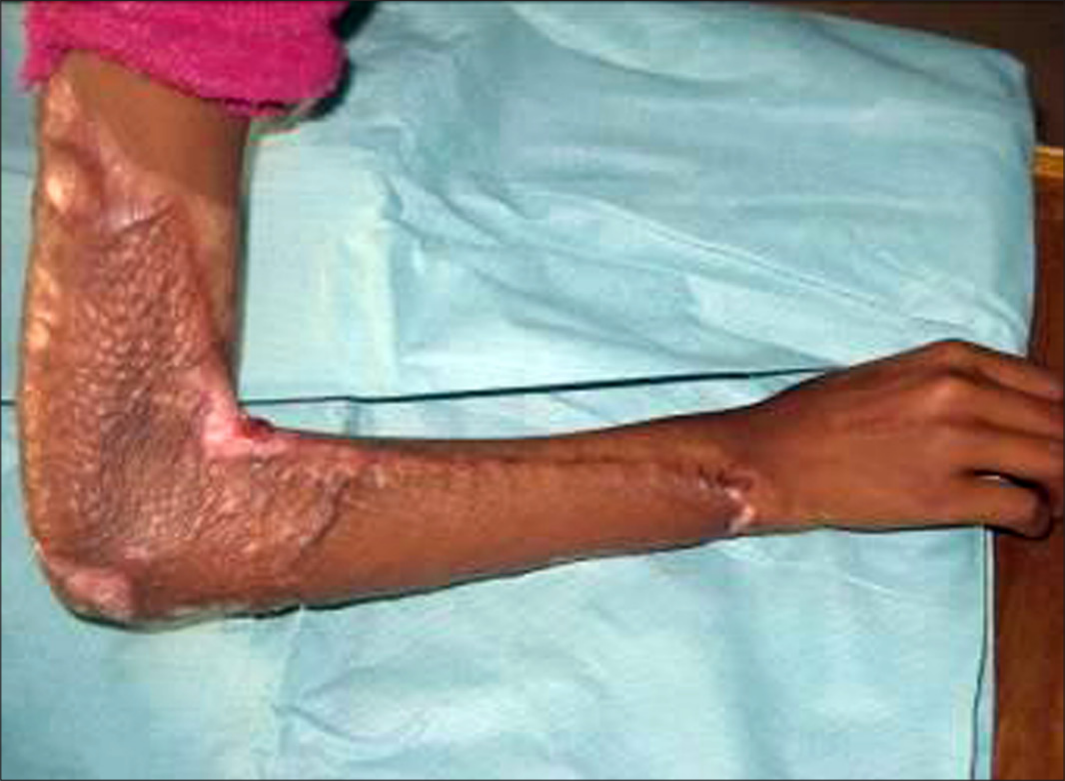
Flexion contracture at the elbow can be avoided by keeping the elbow in extension by an extension splint

**Figure 6:**
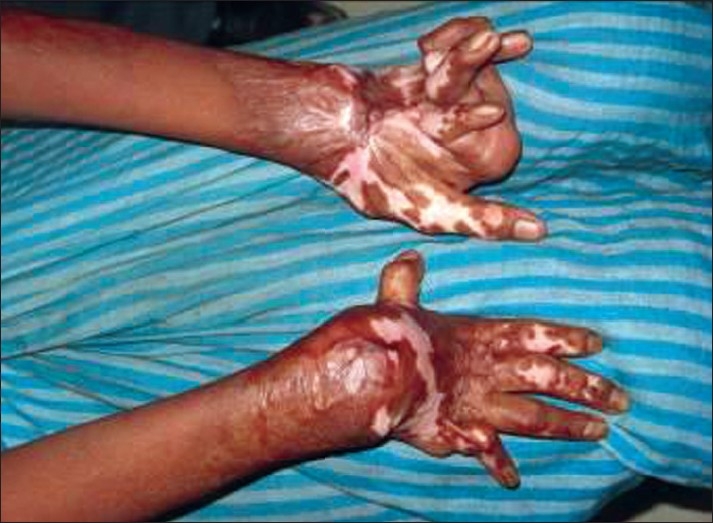
Clawing of fingers can be avoided by keeping the MP joints in flexion, IP joints in extension, thumb mid palmar radial abduction

**Figure 7:**
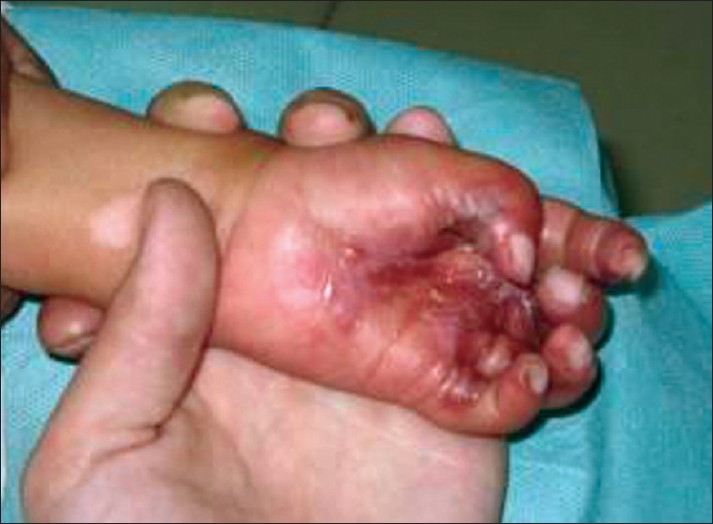
The thumb in palm deformity is avoided by keeping the wrist extended with minimal MCP flexion and keeping the fingers extended and thumb abducted

**Figure 8:**
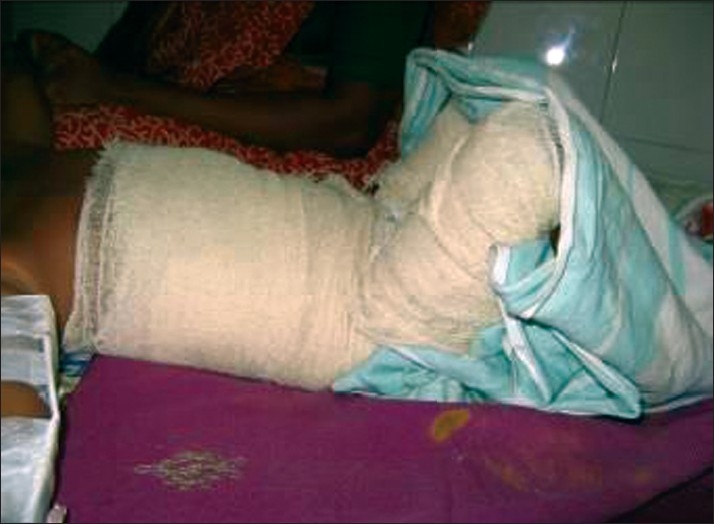
Flexion contracture of the hips can be prevented by lying prone with legs extended. Limit sitting and side lying. Supine lying with legs extended, no pillow under knees. Nursing in this position will cause flexion contractures in the hip and knee joints

**Figure 9:**
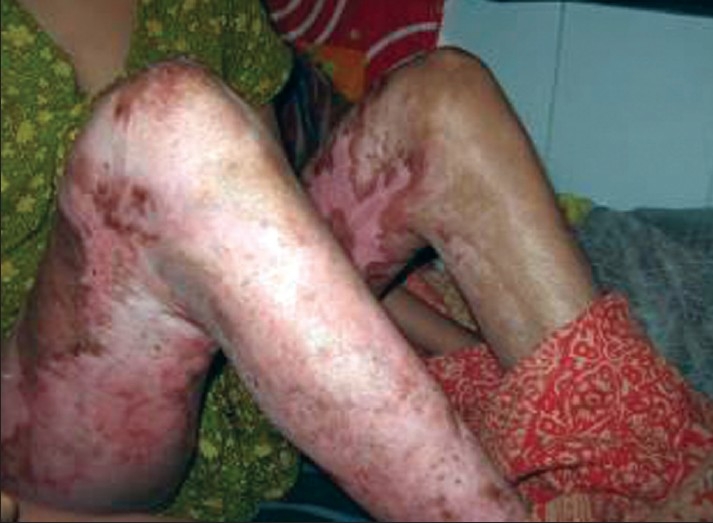
Flexion contracture of the knee can be avoided by keeping the legs extended in lying and sitting and by using knee extension splints

**Figure 10:**
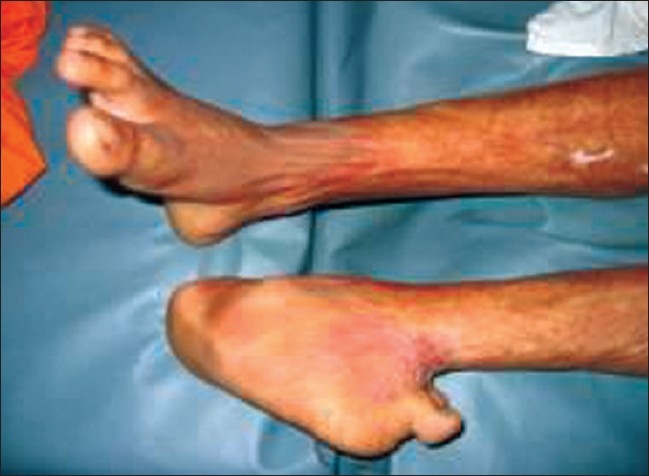
Dorsal contracture at the ankle can be prevented by keeping the ankles at 90 degrees - use pillows to maintain position and encourage sitting with feet flat on floor as long as no oedema is present

**Figure 11:**
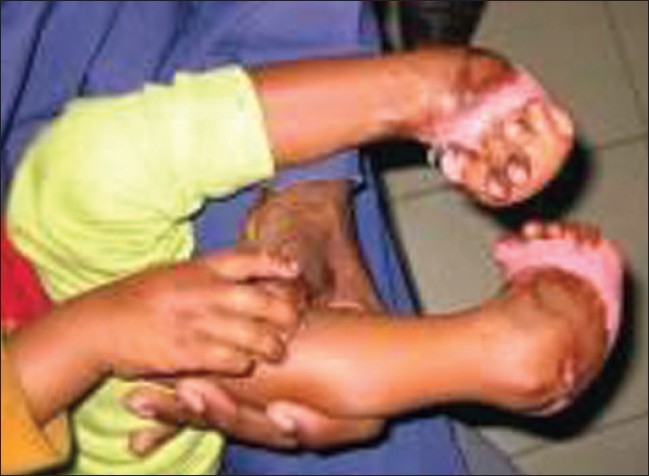
Complex bilateral lower limb contractures which can be avoided by proper anti deformity splintage

Positioning a patient may take some lateral thinking in order to achieve the required position and prevent the patient from gradually relaxing back into a position of contracture.

Use of materials readily available in the ward such as pillows and drip stands (for elevation) can be used as effective positioning tools.

**Figure 12:**
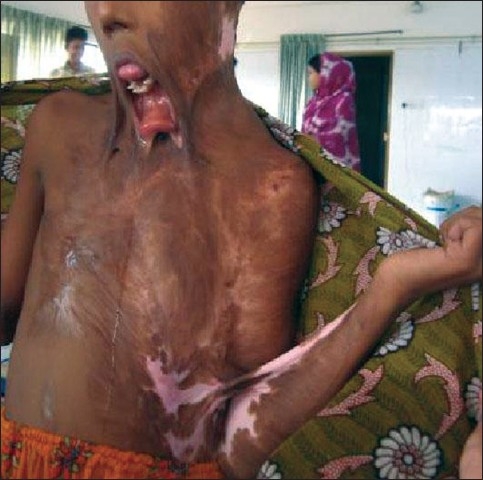
This gross mandibular deformity, malocclusion and neck contracture can be prevented by proper nursing and splintage. A well padded tube can be inserted into the mouth to combat mouth contracture

Simple but consistent positioning from Day 1 can have a significant effect in making contractures avoidable. Many burn contractures can be minimised or avoided completely by early intervention [Figures 11 and 12].

## SPLINTING

Splints are a highly effective method of helping prevent and manage burn contractures and are an integral part of a comprehensive rehabilitation programme.[6] Splinting helps maintain anti-contracture positioning particularly for those patients experiencing a great deal of pain, difficulty with compliance or with burns in an area where positioning alone is insufficient. If the injured site is over joint surfaces, special precautions should be taken to identify all possible joint contractures. A well-designed splintage programme incorporated with active and passive mobilisation is essential to prevent and convert joint contractures and deformities.[1]

Splinting can provide a stretched position, which also provides an easier starting point for exercise and stretching regimes. As scars not only contract but also take the shortest route possible, they often cause webbing across natural concavities and joints for example to the neck, knee and axilla; splints appear to help remodel scar tissue as it forms and maintains the anatomical contours. Splinting is the only available therapeutic modality that applies controlled gentle forces to soft tissues for sufficient lengths of time to induce tissue remodelling.[4] Prevention is always better than cure. Early application of splints to prevent the development of post-burn scar contracture in the acute stage is essential.[1]

Splints can be made of various different materials. The ideal material is low temperature thermoplastic as it is lightweight, easily mouldable and remouldable and conforms extremely well to contours. However, this is not the only material which splints can be made from and is not always readily available, in which case alternatives need to be used and improvisation may be necessary.

## MATERIALS FOR SPLINTING

**Figure 13:**
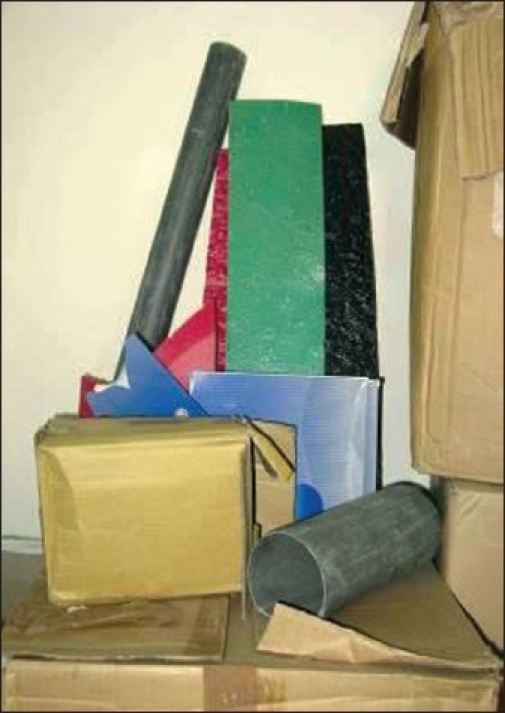
Materials used for making splints

Materials that are readily available are used to make splints [Figure 13]

Plaster of Paris - This material is excellent in the early stages while a patient is immobile and has heavy dressings applied; however, it tends to absorb exudate, is heavy and breaks easily. It is often applied following surgery to immobilise and position a limb; however, once it is discarded it must be replaced by something else.

**Figure 14:**
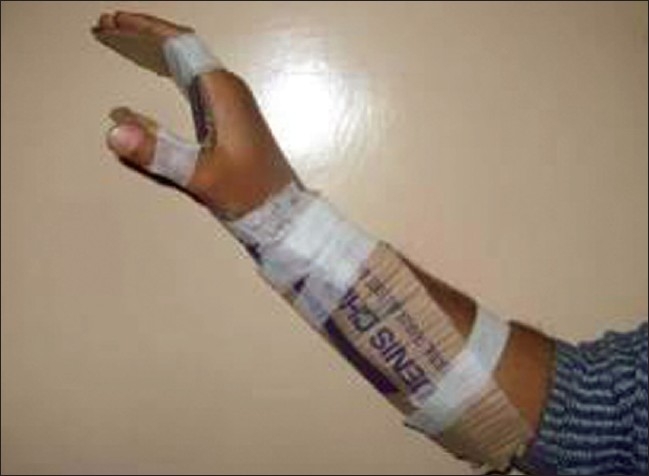
Cardboard hand splints made of discarded dressing boxes

Cardboard - This material also makes an excellent early splint material and is particularly good for positioning and stretching burns to children’s hands. Use of discarded dressing boxes to fabricate easy, lightweight, disposable splints also minimises cost. A dorsal block can be applied over the digits to enhance stretch and a firm (but not too tight) bandage maintains the required position of the hand. Other resting splints can also be made quite simply from cardboard with a little ingenuity [Figure 14].

**Figure 15:**
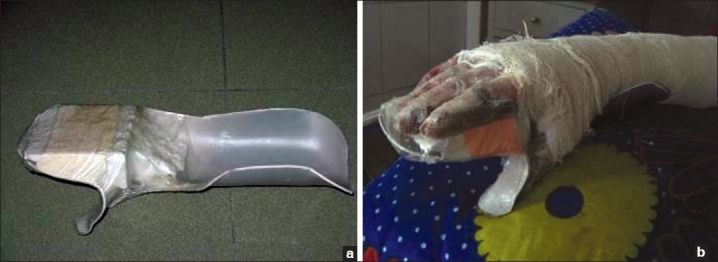
(a,b) Foam and blown polystyrene along with PVC pipes used to make hand splints

Foam and blown polystyrene - These make excellent positioning tools particularly for maintaining position of large joints while the patient is on bed rest, for example, axilla in abduction. They are useful to position patients at night. They can also be used in conjunction with other materials to create splints for example with PVC to create hand splints [Figures 15a–b].

**Figure 16:**
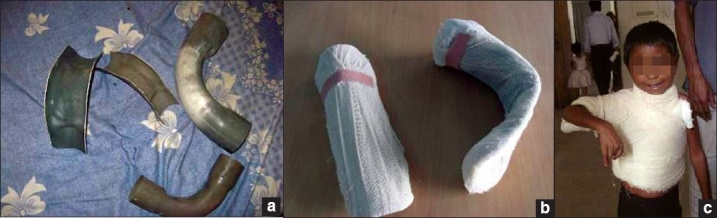
(a) PVC pipes and elbows cut, (b) padded and (c) fabricated into axillary splints

PVC piping - This material is easily made into lightweight, effective splints which can be comfortably worn by patients. It can be cut with a saw and grossly shaped with heavy duty scissors to create knee arm and finger extension splints and with addition of other materials such as foam and blown polystyrene, hand splints can be created. PVC elbow pipes can be cut lengthways, padded and can be worn as very effective axilla splints [Figure 16].

PVC can also be heated (with use of an electric heat gun) and moulded to create splints with similar conformability to those made from thermoplastic materials. Unlike low temperature thermoplastics, it cannot be moulded directly to the patient due to its high melting point, but can be heated and remoulded until a good fit is achieved.

**Figure 17 a and b:**
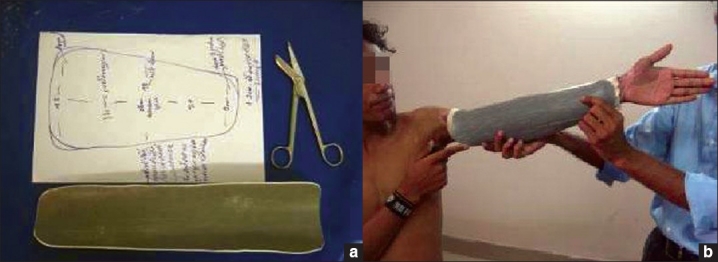
Fabrication of elbow extension splint

**Figure 18:**
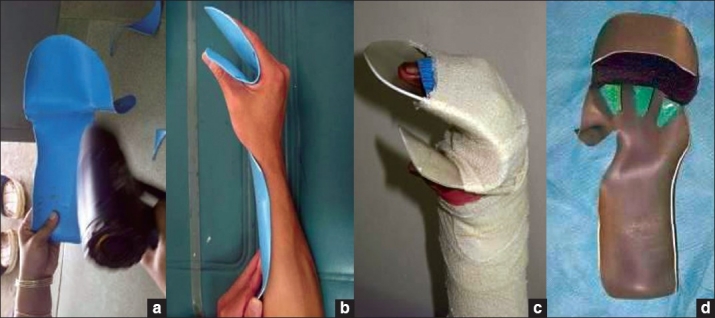
Fabrication of hand splints from PVC sheets

PVC can be used for fabrication of an elbow extension splint [Figure 17]- same method is applied for knee and finger extension splint. Fabrication of a hand splint from PVC [Figure 18] is both easy and comfortable offering excellent patient complaince.

**Figure 19 a - d:**
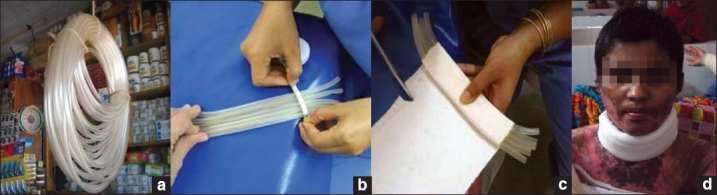
Fabrication of neck splints from garden pipes

Rubber tubing taped together create an excellent neck collar which promotes neck extension, maintains contours and can be worn easily by the patient allowing them to move and function normally [Figure 19].

**Figure 20:**
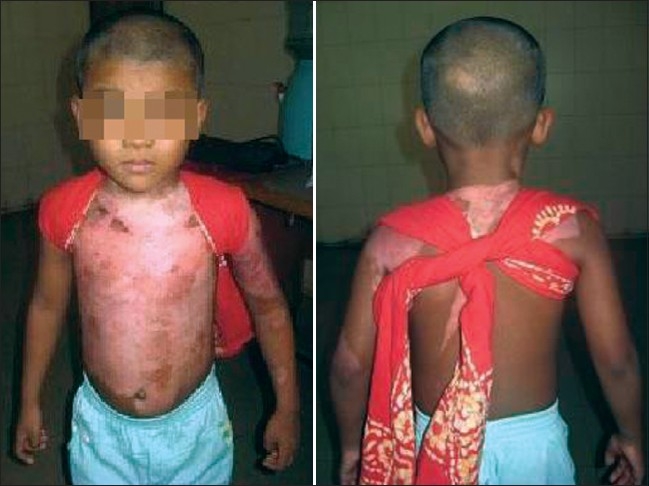
A scarf used for postural support and to stretch pectoral scar

Lengths of material, for example a scarf, make an excellent postural support to stretch scarring to the chest and axillas and can also be used to hold axilla splints in place [Figure 20].

Splinting materials are everywhere around us and with a little ingenuity everyday materials can be used to create effective splinting tools.

## STRETCHING AND EARLY MOBILISATION

Joints affected by burns should be moved and stretched several times a day and the patient is likely to require assistance of members of the burn team and family to reach full range of movement. Therapists use clinical judgement based on the appearance of the tissue as to whether passive range of motion (ROM) or active ROM is performed and also to determine when ROM is resumed after immobilisation.[5] Patients require development of movement habits that are patient specific from day of injury, not when surgery has been completed or dependent on the wound being partially or completely healed.[3] Children may need additional encouragement, therefore the understanding and participation of their parents is crucial from an early stage as they will be helping the child carry out their exercise and stretching regime. Games which incorporate therapy goals such as stretching to catch a ball, reaching and bilateral use of hands depending on the site of injury and therapeutic needs should be encouraged. Pain control is essential to make this process as easy as possible for the patient as it is common for patients to be extremely reluctant and fearful to move if this will cause severe pain. They should be encouraged to mobilise as soon as possible post-injury. Stiffness is common in burn patients both in joints effected by a burn injury and in other joints when immobilised for periods of time. Splinting should be accompanied by regular exercise regimes as contractures can occur, as well, in desirable positions if a patient is persistently splinted and restricted to that position.[6] Patients should be encouraged to get out of bed and exercise as soon as they are fit enough to do so. Therapeutic exercise encompasses ambulation of joints, consideration of neurovascular integrity, improving cardiovascular and respiratory capacity, coordination, balance, muscle strength and endurance, exercise performance and functional capacity.[5] Exercise also helps the patient to experience a general feeling of wellbeing and a sense of confidence and achievement.

Before starting to mobilise a patient, it is important to check there has been no recent surgery, for example skin grafting, or medical issues that contraindicates getting the patient up and moving. Aim to mobilise all patients as soon as possible, if unwell closely monitor vital signs and make continued assessments. If the patient will ultimately not have mobility problems ask relatives to give ongoing support to the patient to exercise. Give consistent encouragement and start slowly, gradually building up the patient’s confidence and exercise tolerance; burn patients often tire quickly so this should be

## ENCOURAGE ACTIVITIES OF DAILY LIVING

Burn patients often feel a sense of loss of role and ability to participate in normal activities of life. Activities of daily living play an extremely important role in a burn patient’s successful outcome. If a patient can accept the responsibility of self exercise and activities of daily living, then the most difficult aspects of rehabilitation are easily achieved.[2] It is crucial to involve patients in daily activities such as eating and washing themselves as soon as possible. Family members should be discouraged from completing these activities for the patient as this emphasises the ‘sick role’ and increases reluctance of the patient to actively participate in their rehabilitation. Highest levels of independence should be encouraged in all activities of daily living from as early as possible.

Participation in their own cares quickly gives the patient an increased sense of wellbeing and control over their environment. Increased ability to perform activities of daily living leads to increase in self-esteem, self-worth and sense of independence and leads to increased motivation levels and desire to improve. Bathing, toileting, feeding, grooming, dressing and vocational skills also incorporate therapeutic goals, for example increased ROM and strength, fine motor and balance. It is important to remember that a child’s vocation is play; children should be encouraged to play and participate in their normal routines as part of their rehabilitation.

## EDUCATION

It is essential that the patient is educated at every stage as to the reasons for the various aspects of their burn rehabilitation and why their participation is crucial to ensure the best possible outcome. Education is of paramount importance along with a consistent approach from all members of the multidisciplinary team.[2] Some individuals will require information to be repeated many times and it is important to make sure they thoroughly understand what they need to do and why. Ongoing education will help the patient take responsibility for their own rehabilitation and in turn help to improve compliance. Initial reluctance due to frustration, pain and fatigue is to be countered by encouragement and education.

## LATER STAGES OF REHABILITATION

### Psychological impact

Excepting the most superficial burns, by definition, we are engaged in treating a chronic condition. There is a groundswell of opinion and literature that strongly supports the use of early intervention and the bio-psychosocial model in other patient populations. This model is particularly pertinent for burns survivors.[3]

Psychological difficulties can occur at any stage following a burn injury. Some individuals find that the impact of the trauma of the initial event may only start to affect them once they are discharged from hospital. Initially the patient may appear to be dealing well with their injury and change in circumstances; however, once the permanence of the situation has become a reality and the longevity of the rehabilitation process is realised, the patient can then start to experience psychological difficulties in the form of depression, anger and anxiety; they may also experience feelings of loss, grieving for their former life, identity and function. If an individual is affected in this way, it is important that they receive the right support and reassurance. Children may show signs of regression in their development and may temporarily become more reliant on their parents than they were prior to their burn injury.

## SCAR MANAGEMENT

Hypertrophic scarring is common following a burn injury and may cause significant functional and cosmetic impairment.[7] The longer a wound takes to heal, the greater the likelihood of hypertrophic scars developing; the risk increases significantly when a wound takes 21 days or longer to heal. Hypertrophic scars are an exaggerated response of the body’s healing process; they have a high blood flow and increased levels of collagen and are extremely active becoming raised, red and rigid. These scars tend to have a high rate of contraction and have other symptoms associated with them including itchiness, dryness and lack of pliability. Hypertrophic scars are generally at their most active for the first 4–6 months post-healing. Initially a scar may appear flat when it is first healed but it is important to monitor scars closely as they may suddenly start to show signs of hypertrophy. It is common for patients to be discharged from hospital with full ROM; however, several weeks later, if corrective measures are not taken to oppose the contractile force of the scar, ROM is lost and scar contracture occurs. Scar management for post-burn injury is a long and often painful process; it is not something that can be carried out for a few weeks and then abandoned, it is something which must continue for many months to minimise post-burn complications from occurring. There is no consensus regarding the best treatment to reduce or prevent hypertrophic scarring;[7] at present little can be done to prevent the formation of scar tissue but a multitude of treatment interventions are used to avert the malady of wound and scar tissue contracture[6] and reduce the impact of the scarring process.

### Positioning

Anti-contracture positioning should continue to be encouraged for many months post-injury whenever the individual is at rest.

### Splinting

Splints prescribed are not only essential for positioning but also for stretching and lengthening the contracted scar tissue.[1] Continued early splintage removed only for exercise and specific functional activities can maximise long-term outcome and can be continued for 6 months post-healing to 2 years or sometimes longer in children. Initially splints are worn for most of the day and night – sometimes for many months depending on scar activity. The splinting regime should be reduced gradually to overnight splinting once ROM is being maintained. The overall length of the scar tissue will be increased by mechanical stretching after application of splintage[1] as splinting is the only therapeutic modality that applies controlled gentle force to soft tissue over a prolonged period of time to cause tissue growth.[4] Continued use of splints helps significantly to stretch scar tissue as it forms, apply pressure to problem areas and maintain anatomical contours - for example of the hands, axilla and neck. A well fitting splint is extremely effective in maximising long-term functional outcome; sometimes compromising function in the short term. Splinting and positioning should always be accompanied by an active exercise and stretching regime. Measurements of range of motion are critical guideposts for defining splint efficacy.[4]

### Stretching and exercise

In the early stages, post-wound healing scars are extremely active and dynamic and the contractile force is at its highest. If the burn is close to or over a joint, it must be stretched to avoid loss of ROM and to prevent a post-burn contracture developing. Preventative and maintenance exercises and splinting programs, employed prior to the development of contractures, are crucial to preserving required functional soft tissue length and glide.[4] Stretching of affected joints several times a day to their maximum functional range, in conjunction with a splinting regime appears to help elongate the scar tissue maintaining ROM. However, if compliance of this regime is not maintained often over many months then the scar will once again contract.

It is important that the individual maintains a good exercise regime, which will also help to stretch scar tissue as well as improving exercise tolerance and maintaining a positive mental state.

### Massage and moisturising

Scar massage is widely advocated as an integral part of burn scar management; while the exact mechanisms of its effects are not known, it appears to help in several ways:

- Application of a moisturiser - burn scars are often lacking in moisture depending on the depth of the injury and the extent of the damage to the skin structures. They can become very dry and uncomfortable and this can lead to cracking and breakdown of the scar. By massaging with an unperfumed moisturiser or oil, the upper layer of the scar becomes softer and more pliable and therefore more comfortable; this also helps to reduce itching which can also be a common problem.
- When scars become thick and raised, they hold additional fluid which reduces their plasticity. Through deep firm massage of the scar using the thumb or fingertips, the effect of this excess fluid can be reduced. Massaging while performing stretches helps to increase ROM of a limb affected by a burn scar.
- Burns scars contain four times more collagen than other scars which is rapidly laid down in whorls and bundles. Deep massage of the scar in small circular movements is thought to help improve with alignment of the scar tissue as it is formed.
- Sensory impairment and changes in cutaneous sensation is common in burn scars. Regular massage and touching of the scars helps with desensitisation of hyper-sensitive scars.
- Psychological factors of individuals having difficulty in coming to terms with having, what they feel is, an unsightly scar can also be reduced by touching the scar and learning to accept how it looks and feels.

### Pressure therapy

Pressure therapy is a primary modality in burn scar management although the clinical effectiveness has never been scientifically proven.[7] Applying pressure to a burn is thought to reduce scarring by hastening scar maturation and encouraging reorientation of collagen fibres into uniform, parallel patterns as opposed to the whorled pattern seen in untreated scars.[2] There is little written evidence around its mechanism but it is thought to create localised hypoxia to the scar tissue reducing blood flow to hyper-vascular scars and therefore reducing the influx of collagen and decreasing scar formation. As soon as the wounds are fully closed and able to tolerate pressure, patients are fitted with pressure garments.[7]

When available, made to measure pressure garments are fabricated for the individual which apply a consistent level of pressure. When made to measure garments are not available, other materials can be used as effective replacements such as ‘tubi-grip’ elastic support bandages, ‘lycra’ swimwear and cycling shorts, sports head and wrist bands, bandages and to small areas breathable tape can be used. Pressure garments appear to help

- reduce scar thickness/lumpiness
- reduce scar redness
- reduce swelling
- relieve itching
- protect newly healed skin/graft
- prevent contractures/ maintain contours

Pressure garments must be applied as early as possible for maximum effect and worn for 23 h removing only for washing and creaming of scars. In hot climates, however, some patients experience difficulties due to heat and humidity in which case the wearing regime may need to be adjusted in order to accommodate more regular removal. If a patient has taken a long time to heal and if they have had skin grafting, they should be provided with a pressure garment as soon as possible post-healing. If they have had an extensive burn and scattered small unhealed areas remain, a pressure garment can be applied with small topical dressings applied beneath it.

### Silicone

Silicone is another modality used to treat hypertrophic scarring. The exact mechanism of action of silicone in the prevention and management of hypertrophic scars is unclear, although it is likely to influence the collagen remodelling phase of wound healing.[7] It appears to soften, flatten and blanch the scar, making it comfortable and improving its appearance.[8]

### Activities of daily living

Individuals should be encouraged to return to their normal daily routines as soon as possible and should re-establish themselves in their roles in life prior to their burn injury as much as they can.

### Social rehabilitation

Following a burn injury some individuals can feel isolated and alone. They may find it difficult to integrate back into society and take up life as they knew it prior to their injury. They may feel like they are the only one who has suffered such an injury and they may not know how to re-enter society, particularly if they have visible burns scars. These individuals should be encouraged in order to re-establish themselves in their social and vocational lives as soon as they are able to, and their family members should be encouraged to promote this behaviour. For children this will mean re-entering school as soon as they are ready to do so, meeting up with friends and participating in activities and sports which they enjoy. Sometimes relatives can become very protective of the individual, fearing that something may happen again; in their desire to care for and protect the individual to keep them safe, they can sometimes impede the reintegration process. Life after a burn injury, particularly a major injury can take some significant adjusting to however with the right support and rehabilitation, burn injured patients can lead a full life.

## CONCLUSION

Rehabilitation from a burn injury is a lengthy process, which starts on day one and involves a continuum of care through to scar maturation and beyond. It involves a dedicated multidisciplinary team of professionals and the full participation of the patient. Sustaining a burn injury, however big or small can have a dramatic affect on the individual’s physical and psychological well-being and requires teamwork and commitment to help each individual overcome the difficulties they may encounter. While the path is not always easy, with the right support and therapeutic intervention, the commitment of the team to not accept even one contracture,[3] and provide understanding of the psychological and social challenges, the patient can reach their maximum physical, psychological and functional outcome.

## References

[1] Kwan M, Kennis W (2002). Splinting Programme for patients with Burnt Hand. Hand Surg.

[2] Edgar D, Brereton M (2004). ABC of Burns Rehabilitation after burn injury. Br Med J.

[3] Edgar D (2009). Active Burn Rehabilitation Starts at Time of Injury: An Australian Perspective. J Burn Care Res.

[4] Fess EE, McCollum M (1998). The Influence of Splinting on Healing Tissues. J Hand Ther.

[5] Richard R, Baryza MJ, Carr JA, Dewey WS, Dougherty ME, Forbes-Duchart L (2009). Burn Rehabilitation and research: Proceedings of a Consensus Summit. J Burn Care Res.

[6] Richard R, Ward RS (2005). Splinting Strategies and Controversies. J Burn Care Rehabil.

[7] Bloemen MC, Van der Veer WM, Ulrich MM, Van Zuijlen PP, Niessen FB, Middelkoop E (2008). Prevention and Curative Management of Hypertrophic Scar Formation. Burns.

[8] Smith FR (2005). Causes of and treatment options for abnormal scar tissue. J wound Care.

